# Dual lag screw cephalomedullary nail versus the classic sliding hip screw for the stabilization of intertrochanteric fractures. A prospective randomized study

**DOI:** 10.1007/s11751-012-0146-3

**Published:** 2012-10-20

**Authors:** G. Kouvidis, V. I. Sakellariou, A. F. Mavrogenis, J. Stavrakakis, D. Kampas, J. Galanakis, P. J. Papagelopoulos, P. Katonis

**Affiliations:** 1 Department of Orthopaedic Surgery and Traumatology, University of Crete, Heraklion, Greece; 2 1st Department of Orthopaedic Surgery and Traumatology, Medical School, University of Athens, Chaidari, Greece; 3 9-11 Antoniadou Street, Nea Smirni, 17123 Attica, Greece

**Keywords:** Dual lag screw intramedullary nail, Sliding hip screw, Intertrochanteric fractures

## Abstract

This study is a randomized prospective study comparing two fracture fixation implants, the extramedullary sliding hip screw (SHS) and the dual lag screw cephalomedullary nail, in the treatment of intertrochanteric femoral fractures in the elderly. One hundred and sixty-five patients with low-energy intertrochanteric fractures, classified as AO/OTA 31A, were prospectively included during a 2-year period (2005–2006). Patients were randomized into two groups: group A included 79 hip fractures managed with sliding hip screws and group B included 86 fractures treated with cephalomedullary nails. Delay to surgery, duration of surgery, time of fluoroscopy, total hospital stay, implant-related complications, transfusion requirements, re-operation details, functional recovery, and mortality were recorded. The mean follow-up was 36 months (24–56 months). The mean surgical time was statistically significantly shorter and fluoroscopy time longer for the group B. No intraoperative femoral shaft fractures occurred. There was no statistically significant difference in the functional recovery score, reoperation, and mortality rates between the 2 groups. A new type of complication, the so-called Z-effect phenomenon, was noticed in the cephalomedullary nail group. There are no statistically significant differences between the two techniques in terms of type and rate of complications, functional outcome, reoperation and mortality rates when comparing the SHS and the cephalomedullary nail for low-energy AO/OTA 31A intertrochanteric fractures. Our data do not support recommendations for the use of one implant over the other.

## Introduction

The incidence of fractures of the proximal femur shows an increase as the population ages. It is estimated that 1.26 million hip fractures occurred in adults in 1990, with predictions of numbers rising to 7.3–21.3 million by 2050 [1]. These fractures are an economic burden because they occur in patients with co-morbidities which influence the quality of life of the patients and also increase the cost of treatment for the health systems. Prompt surgical fixation and fast-track rehabilitation programs have been adopted to facilitate rapid recovery, mobilization, and decrease the intraoperative and postoperative complications [2–4]. One-year mortality varies from 12 to 37 % [5] with about 9 % of these deaths being directly attributed to the hip fracture [6]. Among the survivors after a hip fracture, 10–20 % will require adaptation for a more dependent lifestyle [7].

The sliding hip screw has been a gold standard of treatment for low-energy intertrochanteric fractures with good results overall. However, fracture collapse, medialization of the femur, and limb shortening are the known complications related to this type of fixation. Cephalomedullary nails are biomechanically superior for load transfer and have a biological advantage as minimal invasive techniques can be used for implantation; both advantages are thought to relate to a shorter healing and recovery times with improved functional outcome. There is, however, a risk of iatrogenic fracture, additional fracture comminution during nail insertion, and of suboptimal closed fracture reduction [8–12].

The purpose of this prospective randomized study was to compare a new dual lag screw cephalomedullary nail with the classic sliding hip screw for the treatment of low-energy extra-capsular fractures of the hip in terms of surgical time, blood loss, intraoperative and postoperative complications, reoperation, and mortality rates.

## Materials and methods

From January 2005 to December 2006, one hundred and ninety-eight patients with 198 extra-capsular hip fractures were admitted to our trauma unit. All hip fractures of the low-energy AO type 31-A were included. Patients younger than 65 years, multi-trauma patients, patients with previous ipsilateral hip or femur surgery possibly affecting functional outcome, and patients with pathological fractures were excluded. Thirty-three patients were excluded; 13 patients were too frail for any operative intervention, 7 were unable to walk before fracture, 4 had pathologic fractures due to metastatic disease, 3 were under 65 years of age, and 6 patients declined to participate in the study. Finally, one hundred and sixty-five patients (165 fractures) were enrolled and randomized by sealed envelope for treatment with either a sliding hip screw (79 fractures) or the dual proximal screw cephalomedullary nail (86 fractures).

### Fracture fixation devices

**Fig. 1:**
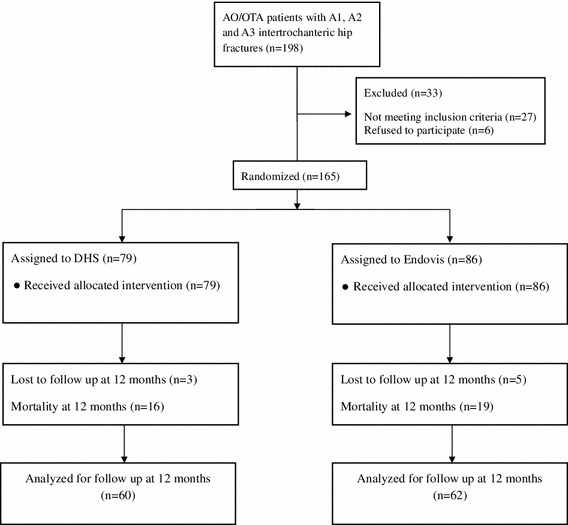
Showing the number of patients enrolled and randomized to the study, the drop outs, and the total number of patients that were followed up

The standard Endovis^®^ Cephalomedullary Nail (Citieffe, Bologna, Italy) developed for the treatment of intertrochanteric fractures is a single sized titanium alloy design which features a cervico-diaphyseal angle of 130°, a metaphyseal angle of 5°, and a total length of 195 mm including 30 mm distal fluted section. The proximal (metaphyseal) diameter of the nail is 13 mm and the distal (diaphyseal) 10 mm. There are two holes for insertion of cephalic screws and one for a distal locking screw. The cephalic lag screws have a shaft diameter of 7.5 mm and an outer thread diameter of 9.7 mm. They also have a self-drilling and self-tapping screw tip design. The distal screw is available in four length sizes, 5 mm diameter, and is self-taping (Fig. 1).

The classic sliding hip screw was first introduced in 1956 for intertrochanteric, peri-trochanteric, and subtrochanteric fractures. We used either the keyed (CLASSIC) or key-less (AMBI) systems in angles 130–140° with 2–4 slots (Smith & Nephew Co.).

### Operative technique

**Fig. 2:**
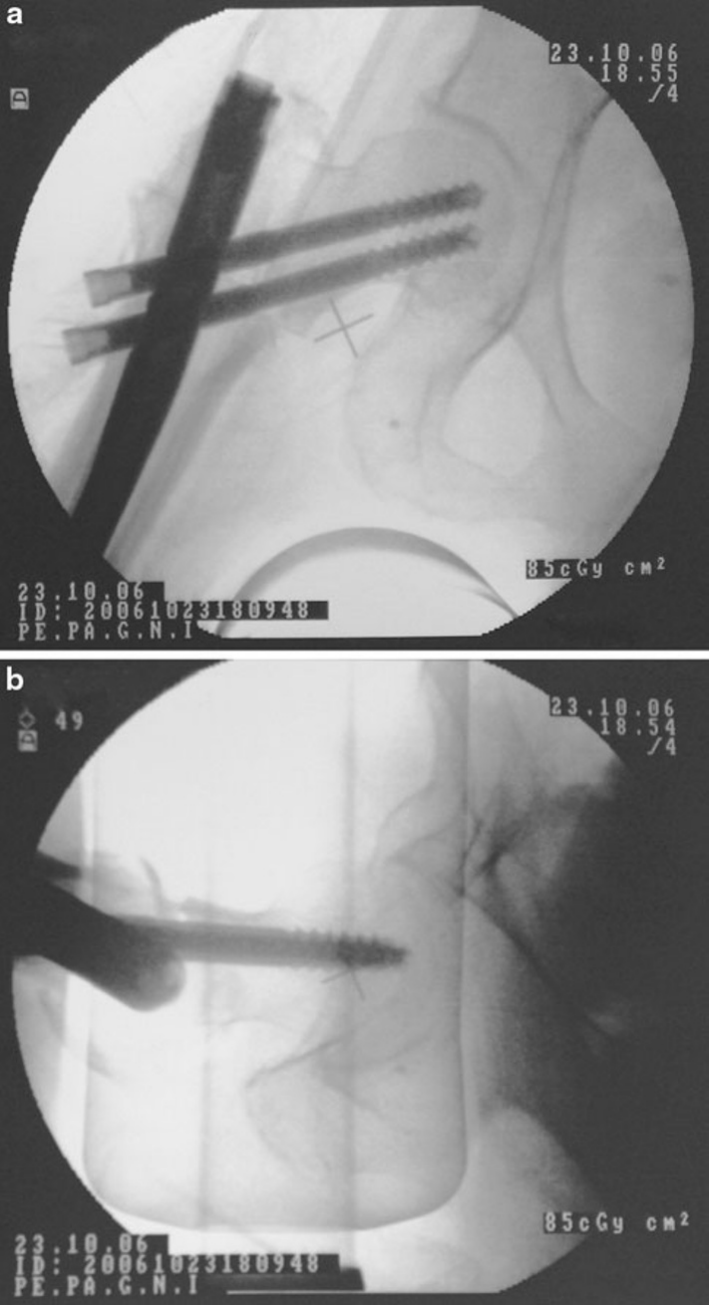
Placement of the distal cephalic lag screw below the midline of the femoral neck, close to or even onto the medial cortex so that the proximal screw will be placed in the center of the head in anteroposterior (**a**) and lateral (**b**) images

The procedures were performed on a fracture table under spinal anesthesia. A closed reduction of the fracture was achieved and documented with the use of an image intensifier. A small lateral approach was utilized. Typically, the trochanteric entry point was identified and the nail was gently advanced to its desired position. In ten cases with a narrow femoral canal, we used an 11-mm-flexible reamer before insertion of the nail. The optimal position for the distal cephalic lag screw is distal to the midaxis of the femoral neck, close to or even onto the medial cortex so that the proximal screw is placed in the center of the head in anteroposterior and lateral images (Fig. 2). The nail was locked distally in all fractures.

### Preoperative and postoperative data

Age and gender, type of fracture, functional status, and surgical risk defined by the American Society of Anesthesiologist (ASA) classification (I–V) [14] were recorded preoperatively (Table 1). The fractures were classified on the basis of OTA/AO classification [13]. Functional Recovery Score (FRS) was used to assess the preoperative and postoperative functional status and mobility [15, 16]. Delay to surgery, total duration of surgery, duration of fluoroscopy, number of blood units transfused, and implant-related complications were recorded postoperatively. Total hospital stay from admission to discharge was also recorded.

**Table 1:** Preoperative data of the patients

	Group I DHS (*N* = 79)	Group II Endovis (*N* = 86)
Sex		
Women	49 (65.4 %)	72 (80 %)
Men	26 (34.6 %)	18 (20 %)
Average age (years)	82.53 (±6.79)	81.95 (±7.21)
Anesthesia risk (ASA)		
I, II	27	31
III, IV	52	55
Functional recovery score (FRS)	84.05 (±15.25)	85.43 (±16.69)
AO/OTA classification	*n* (%)	*n* (%)
(stable A1)	21 (26.58)	26 (30.23)
(unstable A2, A3)	58 (73.42)	60 (69.77)

We used the tip-apex distance to assess differences in position of the implants. Tip-apex distance is the sum of the distance from the tip of the lag screw to the apex of the femoral head on an anteroposterior radiograph and this distance on a lateral radiograph, after controlling for magnification. For the SHS, we used the tip of the sliding screw as a point of measurement, while for the dual screw cephalomedullary nails, we used the tip of the proximal screw as a point of measurement.

### Hospital course

The standard postoperative protocol included an immediate start of passive exercises, and during the first postoperative day, the patients were allowed to begin active lower limb movements and sit on the side of the bed. On the second postoperative day, they were encouraged to mobilize with a walking frame and bear weight as tolerated.

### Follow-up protocol

The patients were re-examined in the hospital at 3 weeks and 4 months postoperatively. At 3 weeks, skin sutures were removed and wound or other complications were evaluated. The functional status was noted. At 4 months, fracture healing and the state of the implant were assessed on X-rays and the progress of functional recovery evaluated using the FRS form. At the end of each postoperative year, patients were contacted by phone and were requested to fill the FRS questionnaire and send new X-rays of their hip.

### Statistics

All data were tabulated in an Excel sheet and were analyzed using the SPSS (version 18) statistical package for personal computers. The Wilcoxon rank sum test and the student’s *t* test were used for ordinal and quantitative variables, respectively, to discriminate differences between two groups. Significance levels were set at *p* < 0.05.

## Results

The distribution of patients after randomization is shown in Table 1. There was no statistically significant difference regarding the age, gender, fracture classification, ASA score, and preoperative functional level between the groups (*p* = 0.89).

The mean duration of surgery for the SHS group was 8 % longer than that for the nail and averaged 55.18 min (SD value = 11.5) for the SHS group and 51.22 (SD value = 12.94) minutes for the cephalomedullary nail group (*p* = 0.03). On the other hand, the intra-operative fluoroscopy time was 33 % shorter for the SHS group; 0.98 (SD value = 0.54) minutes for the SHS versus 1.2 (SD value = 0.74) minutes for the cephalomedullary nail group (*p* = 0.02) (Table 2).

**Table 2:** Operative details

	DHS (*n* = 79)	Endovis (*n* = 86)	*p* value
Preoperative delay^a^	3.18 (2.46)	3.24 (2.44)	NS
Total hospital stay^a^	8.16 (3.24)	9.01 (3.16)	NS
Surgical time^b^	55.18 (11.50)	51.22 (12.94)	0.03*
Fluoroscopy time^b^	0.98 (0.54)	1.2 (0.74)	0.02*
Transfused data	41pts [75 un] 1.05/pt	40 pts [72 un] 0.84/pt	NS

^a^Preoperative delay, and total hospital stay in days, mean (SD)

^b^Surgical time, and fluoroscopy time in min, mean (SD)

* Significant *p* = 0.05

There was no statistically significant difference in the transfusion requirements between the two groups. Specifically, a mean of 1.05 (range 0–2) blood units were transfused in the SHS group while a mean of 0.84 units (range 0–2) were transfused in the nail group (*p* = 0.84, Table 2). There was no statistically significant difference on the mean preoperative delay (*p* = 0.78) or the total hospital stay of both groups (*p* = 0.87, Table 2).

**Fig. 3:**
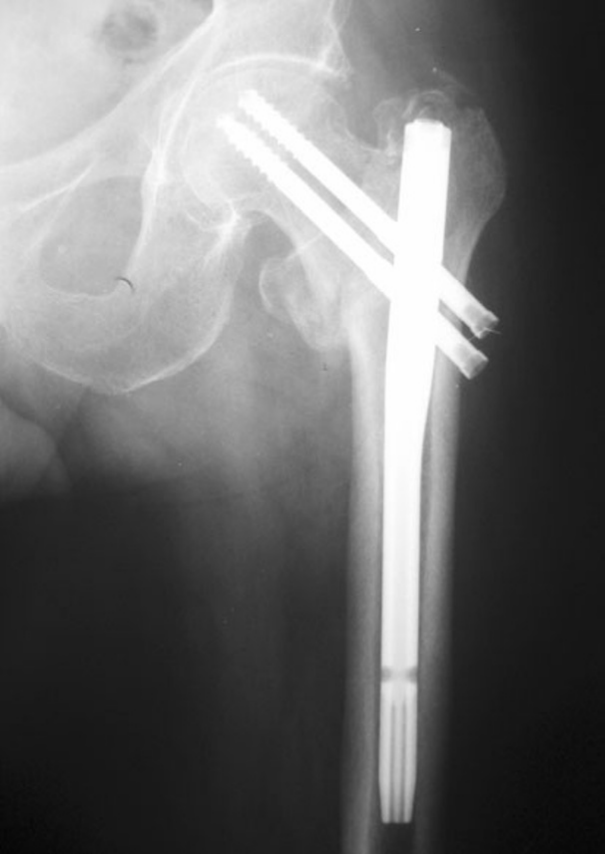
X-ray image showing a typical fracture with acceptable closed reduction, which eventually showed radiographically evident healing at the 4-month postoperative visit

All fractures had an acceptable closed reduction, and fracture healing was evident in all cases by the fourth month on X-rays (Fig. 3). The average tip-apex distance was 24 mm (range 8–59 mm) for the SHS group and 26 mm (range 10–62 mm) for the cephalomedullary nail group (*p* = 0.892).

**Fig. 4:**
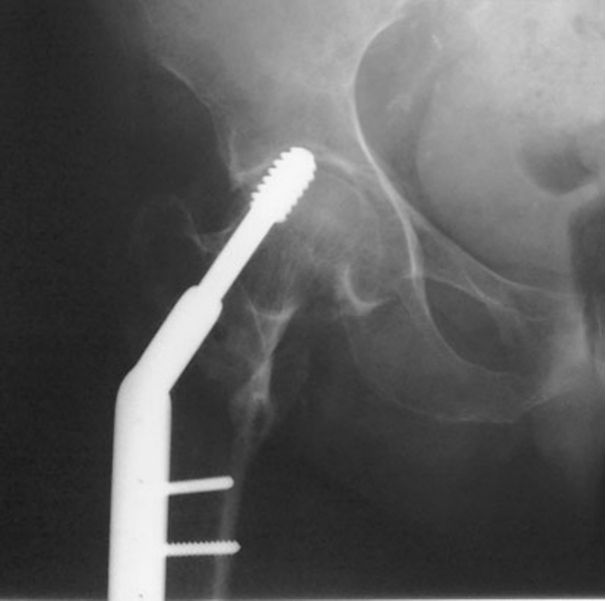
Cutout of the sliding hip screw, which was revised using a new SHS 3 months after the initial operation

**Fig. 5:**
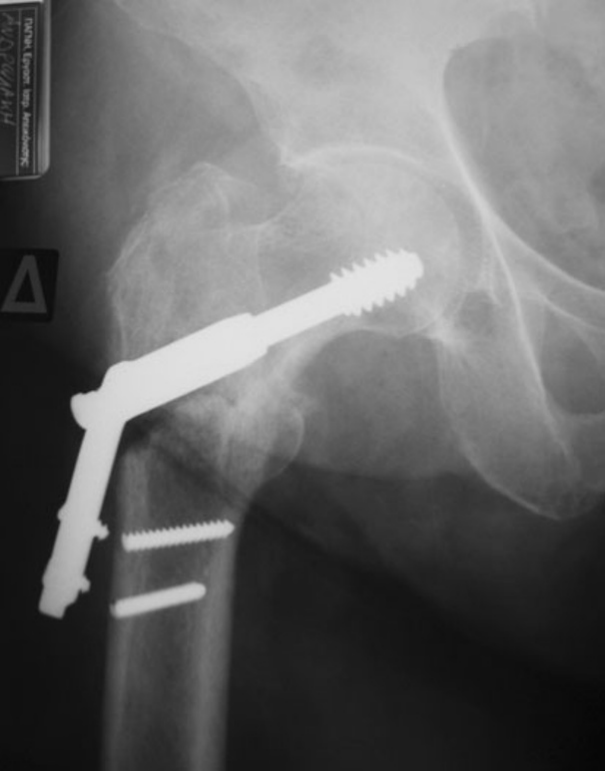
X-ray showing the barrel-plate pulled off the femur as a result of a fall 4 months postoperatively

Complications occurred in 9 cases (11.39 %) of the SHS group and 8 cases (9.3 %) of the IM nail group (*p* = 0.65). Five cases in the SHS group required reoperation due to lag screw cutout. In two of them, a new SHS was applied 2–3 months after the initial operation (Fig. 4). Only removal of the implants was required for the three other cases as the fractures had been already healed (Table 3). In a sixth case, the barrel-plate pulled off the femur following a fall on the ground 4 months postoperatively (Fig. 5). This case was revised using a longer SHS with a longer 4-hole plate.

**Table 3:** Re-operation details

	DHS	Endovis
Lag screw cutout	5	3
Femoral shaft fracture	0	1
Plate pull-off	1	0
Screw back-out	0	3
Total	6	7

**Fig. 6:**
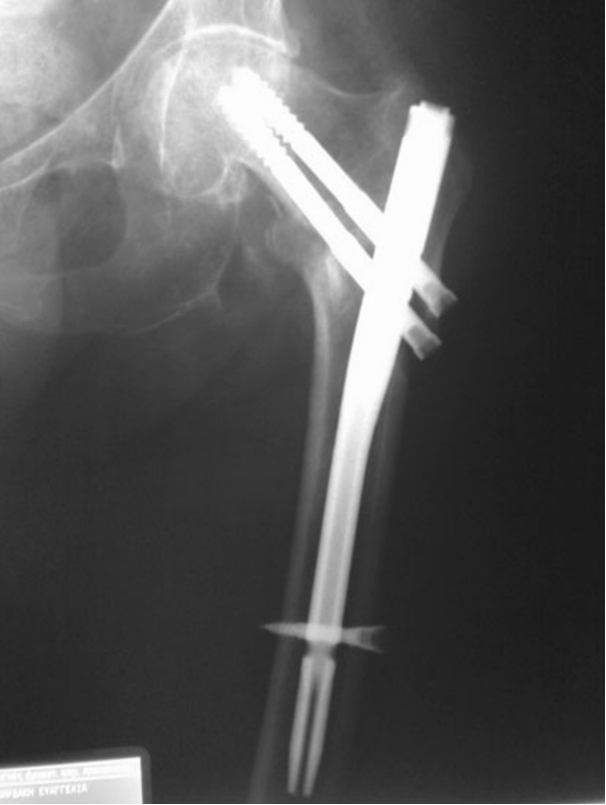
X-ray showing a case in which distal locking screws were missed. The screws left in place and the postoperative protocol were followed as usual without any further complication

In the cephalomedullary nail group, different types of complications were observed. There were two intraoperative fractures of the greater trochanter which occurred during nail insertion. A fracture propagation beginning from an occult fracture line of the greater trochanter was considered as a possible cause. This complication required no special treatment and did not affect the final outcome. In two cases, the distal locking screw missed the nail and was diagnosed only in the postoperative radiograph. These screws were left in situ and the postoperative protocol was followed as usual without any further complication (Fig. 6).

No intraoperative femoral shaft fracture was encountered in this study. There were three cases of lag screw cutout in the IM nail group (Table 3). The intramedullary nail was changed to a SHS in one case. For the other two cases, the proximal screw was removed under local anesthesia. Lag screw back-out occurred in three patients of this group. In one case, it was the superior, and in two cases, the inferior screws backed out 3–4 months postoperatively. However, fracture healing was not impaired and screws were removed 2 months later under local anesthesia without any further complications. There were no implant fractures in this study.

One periprosthetic fracture occurred at the distal tip of the IM nail 6 months postoperatively, as a result of a simple fall. This fracture was revised with a longer IM nail bypassing the fracture line.

Four cases of superficial soft tissue infections occurred, 3 of them in SHS group. All were treated successfully with debridement and intravenous antibiotic administration.

After a mean follow-up of 36 months (range 24–56 months), eight patients (4.84 %) were lost and another 35 (21.21 %) died (Table 4). The difference in 1-year mortality rate between the two groups was not significant. There was no difference between the 2 groups with regard to activities of daily living and mobility at 4 and 12 months postoperatively (Table 5).

**Table 4:** Postoperative 12 months mortality and lost to follow-up

	DHS (*n* = 79)	Endovis (*n* = 86)	Total (*n* = 165)
Mortality	16 (20.25 %)	19 (22.1 %)	35 (21.21 %)
Lost to FU	3	5	8 (4.84 %)
Available to review	60	62	122

**Table 5:** Functional recovery score

FRS	PRE Fx	4 months	1 year
DHS	84.05 (±15.25)	63.65 (±20.94)	74.66 (±21.21)
Endovis	85.43 (±16.69)	64.19 (±25.94)	74.33 (±25.19)

**Fig. 7:**
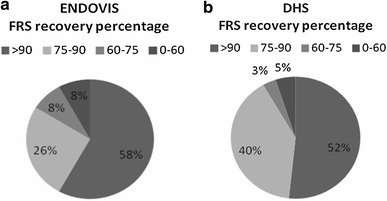
*Charts* showing the patients’ rate for each group that reached more than 90, 75–90, 60–75, and 0–60 % of the pre-fracture mobility level at 1 year postoperatively

Fifty-eight percent of the IM nail group and fifty-two percent of the SHS group achieved more than 90 % of the pre-fracture level status at 1 year. In contrast, 8 % of the IM nail group and 5 % of the SHS group did not achieve independent ambulation and remained in bed or wheelchair. These differences were not statistically significant (*p* = 0.87, Fig. 7).

## Discussion

Intramedullary (IM) nails are increasingly popular compared to plate fixation for treatment of intertrochanteric hip fractures among the young surgeons. A dramatic change in practice has occurred with the intramedullary nail fixation increasing from 3 % in 1999 to 67 % in 2006. This change has been noted despite a lack of evidence in the literature to support this change and potentially known complications [17].

Secondary intraoperative femoral fractures are shown to be the most serious complication related to the standard Gamma nail. Its reported incidence is as high ranges between 0 and 17 % [18–20]. Robinson et al. [21] estimated that incidence of secondary femoral fractures in patients treated with a standard Gamma nail was 18.7 fractures per 1,000 person-years in contrast to the rate of 4.4 per 1,000 person-years with a SHS. In a recent meta-analysis of 25 relevant randomized trials from 1991 to 2005, Bhandari et al. [22] found that intramedullary nails increased the risk of femoral shaft fracture by 4.5 times compared with a compression hip screw. However, among the most recent studies (2000–2005), intramedullary nails did not significantly increase femoral shaft fracture risk. They concluded that previous concerns about increased femoral shaft fracture risk with Gamma nails have been resolved with improved implant design and improved learning curves with the device. In most prior studies, first generation intramedullary nails were used and had proximal nail diameters of 17 mm, available distal diameters between 12 and 16 mm, mediolateral curvature of 10° and a length of 200 mm. These nails required 2 mm overreaming of the femoral medullary canal for easier insertion and this may have been an explanation of the high incidence of secondary fractures intraoperatively.

Leung et al. [23] published a multicenter trial using a modified nail for Asian people with a length of 180 mm, mediolateral curvature of 4°, proximal diameter of 16 mm, and distal diameters of 11 and 12 mm. This modified design of intramedullary nail was associated with a lower rate of intraoperative and postoperative complications than the standard nail. Utrilla et al. [18] who used a new design of Trochanteric Gamma nail reported that the postoperative complications were similar to those with the SHS, without postoperative femoral shaft fractures as had been reported in association with the standard Gamma nail. There was no intraoperative femoral shaft fracture in our study. This is probably explained by the specifications of the IM nail which does not require reaming or hammering during insertion in the medullary canal. Moreover, the design which has 5° of metaphyseal angle, a total length of 195 mm including 30 mm of a distal fluted section, and smaller proximal and distal diameters may be the reasons for the lower complication rates.

Distal locking with one screw was not used routinely in our study but only for A3 reverse oblique fractures as well as in some unstable A2 types when there was rotational or axial instability. It was the judgement of the senior surgeon after releasing the traction and checking for instability by image intensifier screening. This is also supported by Baumgaertner et al. [24], who showed that the nail should be locked distally (generally with one screw) only if rotational or axial instability was observed after the nail and screw are in place and traction is released, but routinely in A3 AO/OTA fractures.

Two biomechanical studies that directly compared the stability of single and dual lag screw implants used for treatment of intertrochanteric hip fractures have shown favorable results for implants with dual lag screws. Kubiak et al. [25] in the first study found that the two implants showed equivalent rigidity and stability and that the dual lag screw implant had a significantly stronger fixation than the single lag screw one when loaded to failure in an unstable intertrochanteric hip fracture model. In the second study, [26] the fixation strength of the Endovis dual lag screw construct was found to be significantly greater than the classic SHS when multidirectional dynamic forces were used for loading. Additionally, double-proximal-screw cephalomedullary nails demonstrated significantly less rotation compared to the SHS. These findings support Ingman`s assumption that the increased rotational stability of the femoral head fixation established by two proximal screws would decrease femoral head cutout [27]. However, in the clinical setting, these biomechanical advantages are not associated with a decrease in complication rates [28, 29]. Our results similarly show no significant difference in cutout between the two implants. There were 5 cases of proximal screw cutout for the SHS group and 3 cases for the cephalomedullary nails; these were all unstable fractures. Moreover, the presence of dual lag screws has introduced a new type of complication, the so-called “Z-effect” and reverse “Z-effect” phenomena [30, 31]. These are axial migrations of the lag screws forward or backward, one at a time or simultaneously and following the same or, more often, the opposite directions. Characteristic screw migration patterns have been described in the literature as the Z-effect involves the lateral migration of the inferior screw, varus collapse of the fracture, and perforation of the femoral head by the superior screw. The reverse Z-effect involves the lateral migration of the superior screw accompanied by the medial migration of the inferior screw. However, in practice, sometimes only one screw actually migrates during the postoperative weight-bearing period. On reviewing the 3 migrations in this series, it was noted these were not typical Z-effect phenomena. In one case, the superior screw backed out 3 weeks postoperatively, and in the other 2 cases, only the inferior screw backed out 3 months after operation. All these 3 patients had unstable trochanteric fractures with comminution of the medial cortex. The reasons for screw migration observed in some types of fractures are still pending and require further investigation [30]. To prevent the so-called Z or reversed Z effects, Lin [32] emphasized the importance of inserting the inferior lag screw as close as possible to, or even right on, the inferior cortex of the femoral neck in order to achieve better anchoring of the screws to a bony area of increased density, thus preventing screw cutout.

Strauss et al. [33] in their experimental study suggested that in cases of intertrochanteric hip fractures with significant medial cortical comminution, surgeons may wish to avoid the use of a two lag screw intramedullary nail. In our opinion, careful surgical technique as well as selection of patients is important and may reduce the complications with these new implants.

Surgeon experience is a critical factor when comparing a familiar implant with a new one. Because of the universal familiarity with the SHS device, any comparison with a new implant must take account of the significant learning curve effect as a source of potential bias [34]. The vast majority of operations in our study were performed by orthopaedic residents under a senior surgeon’s assistance. The participating residents had almost equal experience with both implants. The senior surgeons had already performed more than fifteen Endovis procedures each prior to this study.

## Conclusion

Overall, there is no clear advantage of one implant over the other. Both can be used successfully for the treatment of 31 AO/OTA intertrochanteric hip fractures in the elderly. The duration of surgery was significantly shorter (*p* = 0.03) for the cephalomedullary nail group but with significantly more time for fluoroscopy (*p* = 0.02). These differences are of little clinical importance and did not affect the final outcome or the complication rate between the two methods. The two lag screws of the cephalomedullary implant do not seem to carry any significant difference in clinical practice as supported by previous biomechanical experimental studies. Furthermore, the risk for the so-called Z-effect phenomenon exists while using with this new implant design.
